# Collocated mixed reality for basic life support training in medical students: a randomised pilot feasibility trial

**DOI:** 10.1016/j.resplu.2026.101383

**Published:** 2026-06-10

**Authors:** David Maiershon, Jonathan Giron, Vered Daitch, Yael Feinstein, Hadas Leibovich-Kot, Shlomit Tovbis, Tohar Levartovsky Ovadiya, Hadar Lavi, Ben Hazan, Maxim Lantsman, Alon Basker, Miri Mizrahi Reuveni

**Affiliations:** a Dina Recanati School of Medicine, Reichman University, Herzliya, Israel; b Clalit Health Services, Tel Aviv, Israel; c Sammy Ofer School of Communications, Reichman University, Herzliya, Israel; d Paediatric Cardiac Intensive Care Unit, Schneider Children’s Medical Centre, Petach Tikva, Israel; e Maccabi-Dent, Tel Aviv, Israel; f Maccabi Healthcare Services, Tel Aviv, Israel

**Keywords:** Basic life support, Resuscitation education, Augmented reality, Mixed reality, Simulation, Feasibility

## Abstract

**Background:**

Simulation-based basic life support (BLS) training often lacks patient-level and contextual realism. Mixed reality (MR) may enrich manikin-based simulation with virtual patient cues while preserving tactile interaction. This study evaluated the feasibility of integrating collocated MR-supported BLS training into undergraduate instruction.

**Methods:**

We conducted a single-centre, 1:1 randomised pilot feasibility trial embedded within an 8-h BLS course. First-year medical students were randomised to MR-supported or conventional training. Feasibility domains included course integration, same-day safety and technical delivery, learner-reported workload, and exploratory MR-specific usability and acceptability feedback. Secondary learner-reported outcomes included self-efficacy, situational motivation, student satisfaction and self-confidence in learning.

**Results:**

Sixty-one students participated (MR *n* = 31; control *n* = 30), of whom 53 completed the post-training questionnaire (MR *n* = 24; control *n* = 29). MR-supported training was delivered within the scheduled course. No technical failures prevented delivery, and no falls, severe discomfort, or headset-related events requiring discontinuation were recorded. Learner-reported workload did not differ significantly between groups (NASA Task Load Index mean difference, MR minus control: 0.63; 95% CI −1.20 to 2.47; *p* = 0.49). Secondary learner-reported outcomes did not differ significantly between groups. Exploratory open-ended feedback from 10 of 31 participants in the MR arm identified areas for refinement, including headset stability, avatar responsiveness, and integration into teaching; views of added value were mixed.

**Conclusions:**

Collocated MR-supported BLS training could be integrated into undergraduate instruction, with no evidence of higher learner-reported workload. Further refinements should precede larger evaluations of objective BLS performance, retention, and transfer.

## Introduction

Basic life support (BLS) is a core lifesaving competency taught early in medical curricula.^1^, ^2^, ^3^ Simulation with physical manikins enables repetitive hands-on practise; however, conventional manikin-only training often lacks environmental and patient-level realism. In such scenarios, key patient cues such as illness severity, skin colour, respiratory effort, and scene context are absent, so learners infer or imagine them while performing hands-on skills. This reliance on imagination may reduce psychological fidelity and limit perceived transfer to clinical care.^2^, ^4^

Extended-reality (XR) approaches, including augmented reality (AR) and mixed reality (MR), have been proposed to visually augment simulation-based training while preserving physical interaction with real objects. According to Azuma’s foundational definition, AR systems combine real and virtual content, operate in real time, and rely on three-dimensional spatial registration between virtual overlays and the physical environment.^5^ Because terminology across XR studies remains inconsistent, precise classification of the evaluated hardware-software configuration is important, particularly in resuscitation education.^6^, ^7^ In the present study, MR refers to a passthrough configuration in which virtual patient features and clinical cues are spatially integrated with a physical training device, enabling learners to perceive contextual information directly within the real task space.^8^ In BLS training, anchoring a virtual patient avatar to a physical, feedback-enabled manikin may help increase contextual realism while preserving hands-on interaction during chest compressions.^9^, ^10^ However, evidence for XR-supported resuscitation education remains limited and heterogeneous, with reported findings varying across learner experience, usability, and short-term educational and performance-related outcomes.^10^, ^11^, ^12^, ^13^ A central feasibility concern is whether adding visual complexity and hardware-related demands increases learner-reported workload. Cognitive load theory emphasises that instructional design should reduce non-essential processing, enabling learners to allocate cognitive resources to schema formation and task performance.^14^ Head‑mounted MR could increase workload through unfamiliar hardware, limited field of view, device instability during physical tasks, and the need to integrate virtual and physical cues. However, well-designed augmentation may also make patient cues more salient and reduce the need for imagination or instructor narration.^13^, ^15^, ^16^ Accordingly, establishing whether collocated MR can be integrated into routine hands-on BLS training without increasing learner-reported workload or creating major usability barriers is an important preliminary step before evaluating performance, retention, or transfer.

Educational innovations in simulation-based health professions education are commonly evaluated in stages, from learner reaction to learning, behavioural transfer, and patient- or system-level impact.^17^, ^18^ Accordingly, this early-phase randomised pilot feasibility trial was embedded within an undergraduate BLS course. The primary objective was to examine the feasibility of integrating collocated MR-supported BLS training into routine undergraduate BLS instruction, evaluated across course integration, same-day safety and technical delivery, learner-reported workload, and exploratory feedback on MR-specific usability and acceptability. Secondary objectives were to compare learner-reported basic resuscitation skills self-efficacy, situational motivation, student satisfaction and self-confidence in learning between groups.

## Methods

This study is reported in accordance with the CONSORT guidelines for randomised pilot and feasibility trials.^19^, ^20^

### Study design and setting

We conducted a single-centre, two‑arm, parallel‑group randomised pilot feasibility trial embedded within a single‑day, 8‑h BLS course at Reichman University, Herzliya, Israel. The course was delivered to first-year medical students in a 4-year graduate-entry medical programme. The course took place on 19 August 2025, and participants were recruited on the same day. Participants were randomised in a 1:1 ratio to MR-supported training or conventional training. Assessments were conducted at baseline and immediately after the course.

### Participants

Eligible participants were students enrolled in the course. Exclusion criteria included formal BLS training within the previous 12 months, musculoskeletal limitations precluding effective chest compressions, or self-reported known conditions or prior severe headset-related discomfort that could affect safe participation. Prior clinical experience was assessed using a yes/no question and included volunteer work or professional experience in a healthcare-related role, such as emergency medical service work or previous employment as a nurse, medic, or paramedic. Participants were also asked to specify the type of prior experience. Technological comfort was self-rated on a 1–5 scale, with higher scores indicating greater comfort.

### Randomisation and allocation concealment

Participants were randomised using a pre-specified, computer-generated sequence implemented in Qualtrics XM (Qualtrics, Provo, UT, USA). Simple randomisation was used. Allocation was concealed until assignment; the platform revealed group allocation only after eligibility confirmation and completion of baseline measures. At the start of the course day, the study was explained to the students, who accessed the Qualtrics survey using a QR code. After providing written informed consent and completing the baseline measures electronically, eligible participants automatically received their group allocation via the platform before the hands-on training condition was delivered.

### Blinding

Given the nature of the intervention, neither the participants nor the instructors were blinded to group allocation. Data were exported with anonymised study IDs and group codes removed. Data analysts conducted the primary quantitative analyses on these anonymised datasets and were only unblinded to group labels after the main models had been specified.

### Interventions

#### Course structure and shared training conditions

All participants attended the same theoretical BLS session together before proceeding to their hands-on training activities. The theoretical component consisted of a 45-min face-to-face lecture delivered in a lecture hall and covering the standard content of the American Heart Association (AHA) BLS Provider Course, including recognition of cardiac arrest, the adult BLS sequence, chest compressions, airway opening, ventilation, and automated external defibrillator use. The course was delivered through an AHA International Training Centre (ITC). The broader 8-h course incorporated instructor-led teaching, demonstrations, video-guided Practise While Watching segments, hands-on skills practise, and skills assessment. Automated external defibrillator use and pad placement were practised under the same conditions for all participants, without differentiation by study arm. For the group-specific chest-compression practise component, participants proceeded to their allocated training condition. This component comprised two consecutive 2-min cycles, separated by a brief pause. Both arms followed the same curricular objectives and used the same Resusci Anne QCPR feedback-enabled manikin (Laerdal Medical AS, Stavanger, Norway). Hands-on practise duration and instructor-to-learner ratios were comparable across groups, with training delivered in groups of no more than 10 students per instructor. The observed instructor-to-learner ratio was approximately 1:8.

MR-supported arm: Participants used a Meta Quest 3 headset (Meta Platforms Technologies, LLC, Menlo Park, CA, USA) operating in passthrough mixed-reality mode. This provided a real-world video feed of the surrounding environment, allowing learners to see the physical room, their own position, and the physical CPR manikin while a virtual patient avatar was overlaid in the same task space.

The MR system was developed in-house by the Advanced Reality Lab at Reichman University and was built in Unity (Unity Technologies, San Francisco, CA, USA) using the Meta XR software development kit and Quest image-tracking functionality. A BLS instructor was involved in the development and review of the training. Initial registration was achieved using a quick response (QR) marker placed on the physical manikin, enabling the avatar to be positioned in alignment with the manikin’s shoulder position. Alignment was subsequently supported by the headset’s inside-out tracking and spatial computing capabilities. The headset rendered a photorealistic virtual patient avatar with visual and auditory cues, including skin colour, respiratory movements, intermittent body movements, and abnormal respiratory sounds. A software-defined chest-and-hand interaction zone enabled a limited event-triggered response. Hand-tracking supported detection of interactions within this zone. After a resuscitation motion was detected over the chest, the avatar’s sounds and animation shifted from intermittent shaking and abnormal respiratory sounds to softer breathing and a resting animation. Compression-quality feedback was provided by the same feedback-enabled manikin system used in both arms; the MR avatar itself did not provide additional performance feedback on compression depth, rate, recoil, or hand-position accuracy.

Conventional arm: Participants in the conventional arm practised on the same feedback-enabled manikin under instructor-led guidance, without a headset, MR overlay, or virtual patient cues.

#### Headset fitting, hygiene, and technical monitoring

Headset fitting and adjustment were completed before the hands-on practise interval and did not reduce allocated hands-on practise time. A non-standard upgraded headset strap was used. Approximately 20 headsets were available, allowing most participants to use an individually assigned headset during training. When a headset was reassigned to another participant, its external contact surfaces were wiped with disinfectant wipes. Given the potential for shared head-mounted displays to act as fomites, cleaning between users was incorporated as an implementation precaution.^21^ Safety, discomfort, technical interruptions, and implementation issues were monitored and documented by the study team during the course day.

**Fig. 1:**
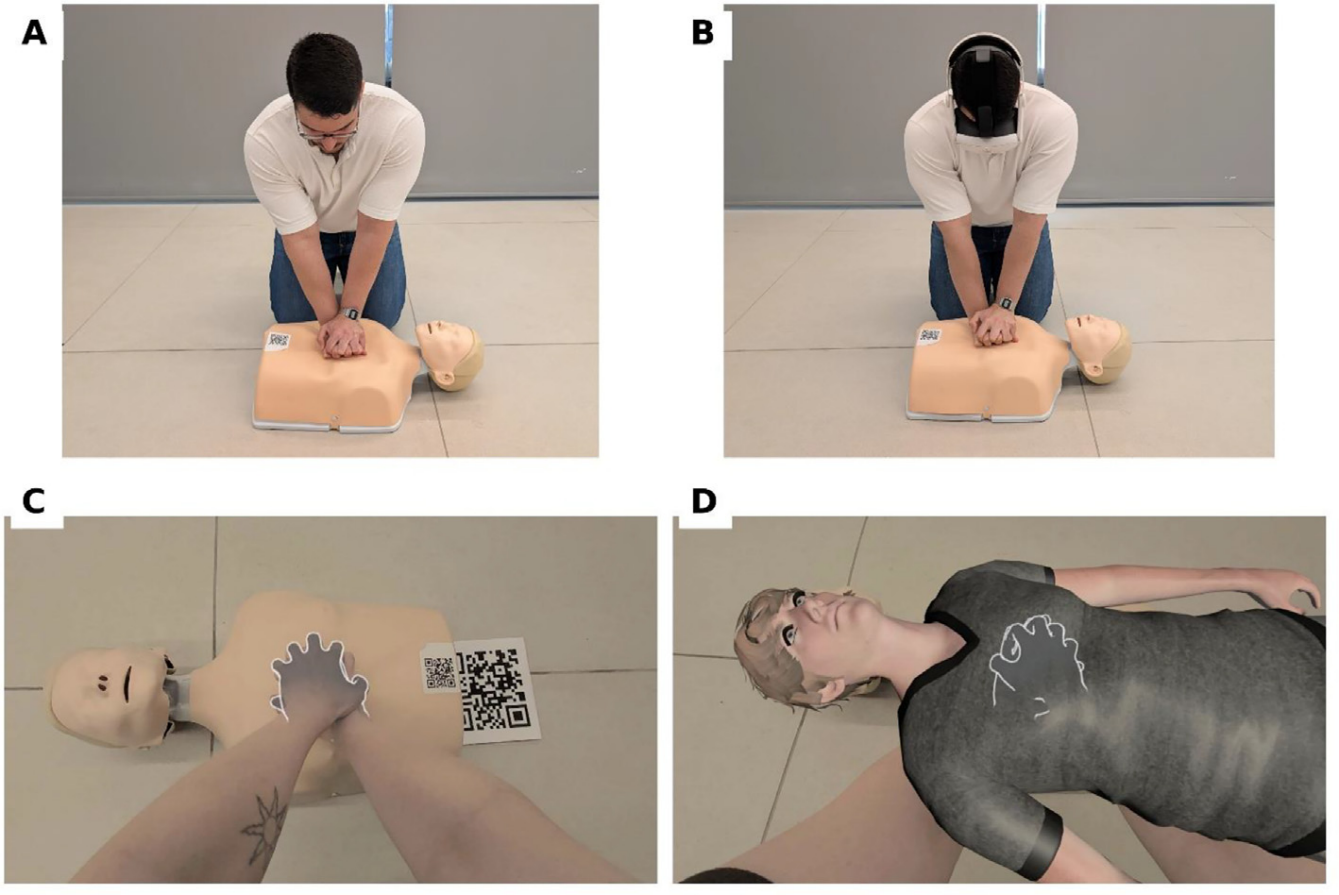
**Conventional and collocated MR-supported BLS training conditions and representative passthrough headset views**. (A) Conventional manikin-only chest-compression practise. (B) Chest-compression practise on the physical feedback-enabled manikin while wearing the Meta Quest 3 headset. (C) Representative passthrough headset view showing the physical manikin, the learner's hands, and the QR image-tracking marker used for initial spatial registration. (D) Representative passthrough headset view showing the virtual patient avatar spatially aligned with the physical manikin within the same task space.

Fig. 1 illustrates the conventional and MR-supported training conditions and includes representative passthrough headset views. During MR-supported training, learners viewed the virtual patient avatar spatially aligned with the physical manikin as they performed chest compressions on the manikin. The MR-supported module was integrated into the BLS skills station and delivered using standardised facilitation. Supplementary Video 1 provides a brief demonstration of the passthrough environment, initial spatial registration using the QR image-tracking marker, and the visual relationship among the virtual avatar, the physical manikin, and the surrounding training space.

### Outcomes and instruments

Learner-reported measures were self-administered electronically through Qualtrics XM at baseline and immediately after training, as applicable. Feasibility was evaluated across four implementation-focused domains: course integration, same-day safety and technical delivery, learner-reported workload, and exploratory MR-specific usability and acceptability feedback. Course integration was assessed by determining whether the MR-supported intervention could be delivered within the scheduled BLS course structure while maintaining comparable hands-on practise conditions across groups. Safety, discomfort, technical interruptions, and implementation issues were monitored and documented by the study team during the course day. Perceived workload was assessed immediately after training using the NASA Task Load Index (NASA-TLX), a widely used, validated, multidimensional instrument suitable for comparing instructional conditions in simulation-based education.^16^ The NASA-TLX score was calculated as the mean of six dimensions, each rated from 0 to 20; higher scores indicated greater perceived workload. A single broad open-ended feedback prompt was administered to participants in both study arms immediately after training. For the exploratory MR-specific usability and acceptability analysis, responses from participants in the MR-supported arm were analysed thematically. The open-ended feedback prompt is provided in Online Resource 1.

Secondary learner-reported outcomes included self-efficacy, assessed at baseline and immediately after training using the Basic Resuscitation Skills Self-Efficacy Scale (BRS-SES).^22^ The BRS-SES score was calculated as the mean of 19 items, with higher scores indicating greater self-efficacy. Motivation was assessed at baseline and immediately after training using the Situational Motivation in Clinical Training scale (SMCIT), a short, study-adapted scale tailored to the immersive BLS training context (provided in Online Resource 2). The post-training SMCIT score was calculated as the mean of two items rated on a 1–5 Likert scale, with higher scores indicating greater situational motivation. Student satisfaction and self-confidence in learning, assessed immediately after training using the Student Satisfaction and Self-Confidence in Learning Scale (SSSCL).^23^ The SSSCL score was calculated as the mean of 13 items, with higher scores indicating greater student satisfaction and self-confidence in learning.

### Sample size

This study was designed as a pragmatic pilot feasibility trial embedded within a required BLS course. The sample size was determined by the number of consenting students in the course and was not powered to detect small between-group differences. Formal progression criteria for a subsequent performance-based trial were not prespecified.

### Statistical analysis

Analyses were predefined for pilot reporting. Descriptive statistics were summarised for baseline characteristics and scale scores. Between-group differences in post-training BRS-SES and SMCIT scores were estimated using ANCOVA, with the corresponding baseline scores included as covariates. Post-training NASA-TLX and SSSCL scores were compared between groups using independent-samples t-tests. Between-group estimates were reported with 95% CIs, together with Hedges’ g as a standardised effect size. Internal consistency (Cronbach’s *α*) was calculated for each instrument. Missing outcome data resulted from non-completion of the post-training questionnaire. Complete-case analysis was used; no imputation was performed. All four quantitative analyses included the 53 participants who completed the post-training questionnaire. Statistical significance was set at *α* = 0.05 (two-sided). All analyses were conducted using IBM SPSS Statistics for Windows, version 28.0 (IBM Corp., Armonk, NY, USA).

### Qualitative analysis

An embedded exploratory qualitative component was conducted using responses to a single broad open-ended feedback prompt administered immediately after training to participants in both study arms. For the MR-specific usability and acceptability analysis, responses from participants in the MR-supported arm were analysed using inductive thematic analysis informed by Braun and Clarke.^24^, ^25^ VD reviewed the responses and generated initial codes. Coding and preliminary theme development were conducted concurrently, with related codes grouped into subthemes and overarching themes as the analysis progressed. Given the limited number and brevity of responses, the analysis was intended to identify preliminary implementation issues and design refinements. Representative quotations were translated from Hebrew and labelled using anonymised participant identifiers. The feedback prompt and a supplementary analytic table presenting themes, subthemes, codes, interpretive summaries, and representative quotations are provided in Online Resource 1. Reporting of the qualitative component was informed by the Standards for Reporting Qualitative Research (SRQR).^26^

### Ethics

The study received ethics approval from the Reichman University Institutional Review Board (039/2025). All participants provided written informed consent. Data were de-identified and stored on encrypted university servers.

## Results

A total of 61 medical students provided informed consent, were randomised, and received their allocated training condition (mean age 29.7 ± 4.5 years; 55.7% were women). Just under half reported prior clinical experience (49.2%), approximately one-third had prior exposure to VR/AR technologies (34.4%), and self-rated technological comfort was moderate (mean 3.4 ± 1.5 on a 1–5 scale, with higher scores indicating greater comfort). Baseline characteristics were similar between the MR-supported training (*n* = 31) and conventional training (*n* = 30) arms, with respect to demographics, prior experience, and technological comfort (Table 1).

**Table 1:** Baseline characteristics of participants by study arm.

**Characteristic**	**MR** **(*n* = 31)**	**Control** **(*n* = 30)**	**Total** **(*n* = 61)**
Age (years), mean ± SD	29.9 ± 4.4	29.6 ± 4.6	29.7 ± 4.5
Women, *n* (%)	17 (54.8%)	17 (56.7%)	34 (55.7%)
Prior clinical experience, *n* (%)	14 (45.2%)	16 (53.3%)	30 (49.2%)
Prior VR/AR experience, *n* (%)	12 (38.7%)	9 (30.0%)	21 (34.4%)
Technological comfort, mean ± SD	3.3 ± 1.5	3.4 ± 1.5	3.4 ± 1.5

Values are mean ± SD or *n* (%) as indicated. MR = mixed reality; AR = augmented reality; VR = virtual reality. Prior clinical experience included volunteer work or professional experience in a healthcare-related role. Technological comfort was rated on a 1–5 scale, with higher scores indicating greater comfort.

### Participant flow and questionnaire completion

Of the 68 students assessed for eligibility, 7 were excluded because they had completed formal BLS training within the previous 12 months. The remaining 61 students provided informed consent, were randomised, and received their allocated training condition: 31 were assigned to MR-supported training and 30 to conventional training. Post-training questionnaires were completed by 53 participants (MR-supported training, *n* = 24; conventional training, *n* = 29). Eight participants did not complete the post-training questionnaire. Post-training quantitative analyses were based on the completed questionnaires. Participant flow is presented in Online Resource 3.

### Feasibility outcomes

The MR-supported training was delivered within the scheduled BLS course structure while preserving the same hands-on practise duration and instructor-to-learner ratio as in the conventional training arm. Headset fitting and adjustment were completed before the hands-on practise interval. During the monitored course day, no falls, severe discomfort, or headset-related events requiring discontinuation were recorded. No technical failures prevented delivery of the MR station. No important deviations from the planned course delivery or allocated training conditions were recorded.

Learner-reported workload did not differ significantly between groups. The between-group difference in post-training NASA-TLX score (MR minus control) was 0.63 (95% CI −1.20 to 2.47; *p* = 0.49; *g* = 0.19; *n* = 53).

### Secondary learner-reported outcomes

**Fig. 2:**
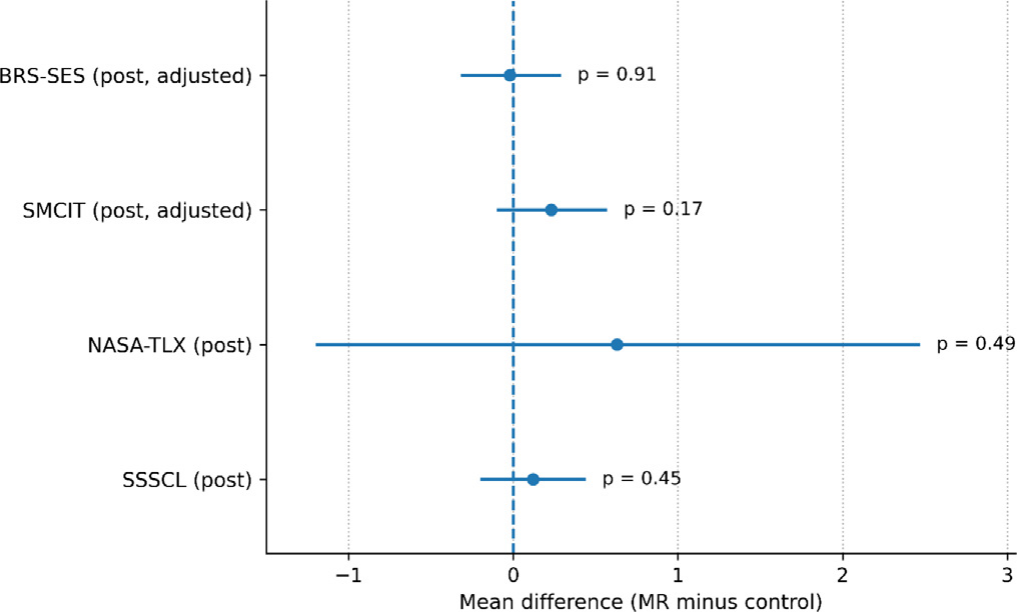
**Between-group mean differences (MR minus control) with 95% CIs for baseline-adjusted post-training BRS-SES and SMCIT scores, post-training NASA-TLX scores, and post-training SSSCL scores**. Points indicate between-group mean differences; horizontal bars denote 95% CIs; the vertical dashed line indicates no between-group difference.

Between-group differences in learner-reported secondary outcomes were small and not statistically significant. The baseline-adjusted between-group difference in post-training BRS-SES score (MR minus control) was −0.02 (95% CI −0.32 to 0.29; *p* = 0.91; *g* = −0.03; *n* = 53). The baseline-adjusted between-group difference in post-training SMCIT score was 0.23 (95% CI −0.10 to 0.57; *p* = 0.17; *g* = 0.28; *n* = 53). The between-group difference in post-training SSSCL score was 0.12 (95% CI −0.20 to 0.44; *p* = 0.45; *g* = 0.21; *n* = 53) (Fig. 2).

### Internal consistency (reliability)

Internal consistency was generally high across the learner-reported instruments. Self-efficacy (BRS-SES) demonstrated excellent reliability (*α* = 0.97; 19 items), and student satisfaction and self-confidence in learning (SSSCL) were similarly strong (*α* = 0.92; 13 items). NASA-TLX yielded *α* = 0.69 across its six dimensions. The brief SMCIT scale yielded lower *α* values (approximately 0.57–0.58), which should be considered when interpreting the motivation findings.

### Exploratory MR-specific qualitative feedback

**Fig. 3:**
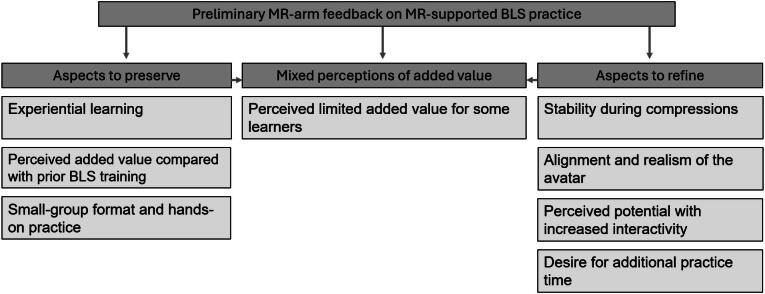
**Preliminary themes derived from exploratory MR-specific open-ended feedback**. The thematic map organises MR-arm feedback into three higher-order categories: aspects to preserve, mixed perceptions of added value, and aspects to refine.

Open-ended feedback was provided by 10 of 31 participants in the MR-supported training arm. The exploratory thematic analysis was organised into three higher-order categories: aspects to preserve, mixed perceptions of added value, and aspects to refine (Fig. 3).

Feedback highlighted practical ergonomic considerations, particularly headset stability during chest compressions. Participants expressed mixed views on the added value of the MR layer: some saw potential in greater interactivity and improved avatar responsiveness, whereas others perceived limited benefit beyond conventional practise. Responses also highlighted the importance of visual and contextual realism, clear integration into the instructional process, and opportunities for further practise. Themes, subthemes, codes, interpretive summaries, and representative quotations with anonymised participant identifiers are presented in Online Resource 1.

## Discussion

This study represents an early-phase feasibility evaluation of collocated MR-supported BLS training embedded within an undergraduate course. The study focused on whether head-mounted MR could be integrated into hands-on resuscitation training, while monitoring course integration, same-day safety and technical delivery, learner-reported workload, and exploratory feedback on usability and acceptability. Despite the additional hardware and the need to integrate virtual and physical cues, we found no evidence that assignment to MR-supported training resulted in higher learner-reported workload than conventional training, as measured by the NASA-TLX. This finding should be interpreted in the context of known constraints of head-mounted MR systems. It should also be interpreted cautiously because the pilot trial was not designed to establish equivalence or non-inferiority. In physically demanding simulation contexts such as BLS training, ensuring alignment between immersive augmentation and task demands represents an important implementation consideration. Future iterations introducing greater interactivity or visual complexity will require careful monitoring of learner-reported workload and related usability outcomes.

Exploratory open-ended feedback from 10 of 31 participants in the MR-supported training arm identified practical areas for refinement, including headset stability during chest compressions, avatar responsiveness, and integration into teaching. Participants expressed mixed views regarding the added value of the MR layer: some highlighted its experiential potential, whereas others perceived limited benefit beyond conventional practise. Beyond immediate usability considerations, participants expressed interest in continued or distributed practise opportunities, highlighting the perceived value of repeated exposure for maintaining confidence and skills over time. Together, these qualitative findings suggest preliminary priorities for subsequent iterations of MR-supported training: optimising ergonomics, enhancing contingent feedback and interactivity, and embedding opportunities for longitudinal practise.

Our findings align with a growing body of literature suggesting that immersive technologies, such as virtual and augmented reality, can be integrated feasibly into BLS and other procedural training contexts. Previous randomised studies have evaluated AR-assisted CPR training using HoloLens-based applications, including real-time guidance and self-training approaches. These studies reported generally comparable chest-compression depth and rate relative to instructor-led training, although findings varied across other performance-related outcomes and educational settings.^27^, ^28^ More recently, a non-inferiority randomised trial among medical students reported that MR-based BLS simulation was non-inferior to manikin-based simulation in terms of overall BLS performance assessed at 1 month.^29^ The present study addresses a complementary implementation question: whether a collocated MR layer spatially aligned with a physical feedback-enabled manikin can be integrated into routine undergraduate BLS instruction while preserving hands-on practise and manikin-based compression-quality feedback. Likewise, recent scoping reviews and meta-analyses highlight substantial heterogeneity across XR modalities, hardware and software configurations, learner populations, and reported outcomes, reinforcing the need to align XR implementation with specific learning objectives.^9^, ^11^, ^30^ Collectively, these findings suggest that immersive formats can be feasible and acceptable in educational settings, although their educational value may depend on learner characteristics and instructional design.

The evidence base for MR-supported BLS training remains limited and heterogeneous, reinforcing the importance of distinguishing implementation feasibility from performance effectiveness. This cautious interpretation is consistent with contemporary resuscitation education guidance. The 2025 American Heart Association guidelines recommend using CPR feedback devices during skills training and advise against using AR as a stand-alone approach for teaching CPR skills. The 2025 European Resuscitation Council guidelines take a cautiously supportive position, suggesting that AR may be used for life-support training when it adds value to the learning process. A recent review of the evolution of AHA, ERC, and ILCOR recommendations similarly highlights that evidence for XR-supported BLS education remains limited and supports cautious integration aligned with clearly defined learning objectives.^31^ In the present study, the MR layer supplemented rather than replaced hands-on practise and compression-quality feedback from the physical manikin system.^32^, ^33^

This study evaluated a specific collocated MR configuration in which a virtual patient avatar was displayed via passthrough functionality and spatially aligned at the task level with a physical feedback-enabled manikin. This configuration allowed participants to perform compressions on a tangible surface while perceiving a virtual patient displaying respiratory movements and sounds within the same physical task space. Prior XR studies in resuscitation education provide an important context for this work, but the evaluated configurations vary in the extent to which virtual content is spatially aligned with a physical manikin during hands-on practise.^34^, ^35^, ^36^, ^37^, ^38^ This configuration combines visual augmentation with preserved physical manikin interaction and is intended to increase contextual fidelity.^15^, ^39^

Strengths of this study include its randomised design within an authentic BLS course, the use of multiple learner-reported measures to assess self-efficacy, workload, and learning experience, and the integration of qualitative feedback to inform design improvements. The incorporation of spatially collocated MR enabled evaluation of this implementation approach within routine undergraduate BLS instruction. Nevertheless, several limitations must be acknowledged. First, this study was intentionally designed as an early-phase feasibility evaluation focusing on implementation and learner-reported outcomes. These outcomes are useful for understanding usability, acceptability, and perceived workload, but they cannot establish whether MR improves psychomotor performance, retention, or behavioural transfer. Second, learner-reported outcomes may in part reflect novelty effects and positive affective responses to immersive technologies. Third, some participants did not complete the post-course questionnaire. Complete-case analysis was used, and the possibility of non-response bias cannot be excluded. Fourth, the exploratory qualitative analysis was based on responses from 10 of 31 participants in the MR-supported training arm to a single broad open-ended prompt and should not be interpreted as an in-depth qualitative evaluation. Fifth, although the avatar provided limited event-triggered responsiveness, it did not provide additional avatar-based feedback on compression quality beyond the feedback provided by the manikin system. The avatar was not designed to reproduce the full range or prevalence of clinical signs observed during real-world cardiac arrest, and refinement of clinically relevant visual cues remains a priority for future iterations. Although no headset-related events requiring discontinuation were recorded during the monitored course day, head-mounted displays may be associated with discomfort and cybersickness.^40^ These outcomes should be assessed systematically using dedicated measures, particularly with longer exposure and more interactive scenarios. Finally, the single-site setting, use of a single MR platform, and pilot sample size limit generalisability and warrant cautious interpretation.

Future research should examine whether enhanced contextual realism translates into measurable improvements in performance, retention, and behavioural transfer across educational contexts. Subsequent iterations of the MR system should incorporate ergonomic and interaction refinements informed by learner feedback. These implementation refinements should be addressed before progressing to a larger performance-based evaluation. Investigating differential effects across learner experience levels may also clarify when, and for whom, immersive augmentation is most educationally beneficial. Beyond BLS, the applicability of collocated MR to other hands-on, context-dependent procedural training scenarios warrants further exploration.

## Conclusions

This randomised pilot feasibility trial suggests that collocated MR-supported BLS training can be integrated into routine undergraduate instruction. We found no evidence that assignment to MR-supported training resulted in higher learner-reported workload than conventional training, while exploratory feedback identified ergonomic, instructional, and interactivity-related refinements. Further studies should assess objective BLS performance, retention, and transfer after these implementation refinements have been addressed.

## Consent for publication

All participants provided consent for the anonymous use of their data for publication purposes. Written informed consent was obtained from the individual shown in Fig. 1 and Supplementary Video 1 for publication of the identifiable images and video. No other identifiable personal information is included in this manuscript.

## Availability of data and materials

The datasets generated and analysed during the current study are available from the corresponding author on reasonable request. Supplementary materials are provided as online resources.

## Authors’ contributions

MMR conceived the overall research idea, supervised the project, and provided guidance throughout study design and execution. JG and DM refined the study concept, developed the methodology, contributed to data interpretation, and provided methodological supervision. ML and BH designed and built the mixed reality system, integrated the software and hardware, and supported technical validation. AB contributed expertise in BLS pedagogy and course implementation. VD coordinated the study, oversaw data collection, performed analyses, and drafted the manuscript. YF, ST, HL, HLK, and TLO assisted with study execution, participant coordination, and data quality assurance. All authors critically reviewed the manuscript and approved the final version.

## CRediT authorship contribution statement

**David Maiershon:** Writing – review & editing, Visualization, Supervision, Methodology, Conceptualization. **Jonathan Giron:** Visualization, Supervision, Methodology, Conceptualization. **Vered Daitch:** Writing – original draft, Writing – review & editing, Project administration, Investigation, Formal analysis, Data curation. **Yael Feinstein:** Writing – review & editing, Investigation, Data curation. **Hadas Leibovich-Kot:** Writing – review & editing, Investigation, Data curation. **Shlomit Tovbis:** Writing – review & editing, Investigation. **Tohar Levartovsky Ovadiya:** Writing – review & editing, Project administration, Investigation. **Hadar Lavi:** Writing – review & editing, Project administration, Investigation. **Ben Hazan:** Writing – review & editing, Validation, Software, Resources. **Maxim Lantsman:** Writing – review & editing, Validation, Software, Resources. **Alon Basker:** Writing – review & editing, Supervision, Methodology. **Miri Mizrahi Reuveni:** Writing – review & editing, Supervision, Methodology, Conceptualization.

## Ethics approval and consent to participate

The study was approved by the Reichman University Institutional Review Board (IRB #039/2025). All participants provided written informed consent before participation. The trial was conducted in accordance with the Declaration of Helsinki and institutional ethical guidelines.

## Funding

This research did not receive any specific grant from funding agencies in the public, commercial, or not-for-profit sectors. The MR system and technical support were provided in-kind by the Reichman University Simulation Centre for research purposes.

## Declaration of competing interest

The authors declare no competing interests.

## Appendix A Supplementary material

Online Resource 1 Preliminary usability, acceptability, and implementation themes derived from open-ended feedback in the MR arm, including subthemes, codes, interpretive summaries, representative quotations with anonymised participant identifiers, and the open-ended feedback prompt.

Online Resource 2 Situational Motivation in Clinical Training scale (SMCIT).

Online Resource 3 Participant flow diagram.

Supplementary Video 1 Brief demonstration of the collocated MR-supported BLS training environment. The video shows the passthrough view of the physical training space, the QR image-tracking marker used for initial spatial registration, and the virtual patient avatar displayed in alignment with the physical feedback-enabled manikin.

## References

[1] Bhanji F., Donoghue A.J., Wolff M.S., Flores G.E., Halamek L.P., Berman J.M. (2015). Part 14: education: 2015 American Heart Association guidelines update for cardiopulmonary resuscitation and emergency cardiovascular care. Circulation.

[2] Cheng A., Nadkarni V.M., Mancini M.B., Hunt E.A., Sinz E.H., Merchant R.M. (2018). Resuscitation education science: educational strategies to improve outcomes from cardiac arrest: a scientific statement from the American Heart Association. Circulation.

[3] Merchant R.M., Topjian A.A., Panchal A.R., Cheng A., Aziz K., Berg K.M. (2020). Part 1: executive summary: 2020 American Heart Association guidelines for cardiopulmonary resuscitation and emergency cardiovascular care. Circulation.

[4] Fijačko N., Abella B.S., Nadkarni V.M., Metličar Š., Agten A.-A., Greif R. (2026). Enhancing virtual reality applications for adult basic life support: insights from a comparative analysis. Virtual Reality.

[5] Azuma R.T. (1997). A survey of augmented reality. Presence: Teleoperat Virtual Environ.

[6] Fijačko N., Metličar Š., Kleesiek J., Egger J., Chang T.P. (2023). Virtual reality, augmented reality, augmented virtuality, or mixed reality in cardiopulmonary resuscitation: which extended reality am I using for teaching adult basic life support?. Resuscitation.

[7] Fijačko N., Štiglic G., Gsaxner C., Chang T.P., Greif R. (2024). Rethinking realities: a call for accurate terminology in eXtended reality studies. Resusc Plus.

[8] Barsom E.Z., Graafland M., Schijven M.P. (2016). Systematic review on the effectiveness of augmented reality applications in medical training. Surg Endosc.

[9] Fijačko N., Metličar Š., Janžekovič B., Abella B.S., Nadkarni V.M., Chang T.P. (2025). Extended reality technologies in adult basic life support education: a scoping review. Resusc Plus.

[10] Ricci S., Calandrino A., Borgonovo G., Chirico M., Casadio M. (2022). Virtual and augmented reality in basic and advanced life support training. JMIR Serious Games.

[11] Dubreucq E., De La Vega S.B., Bouaoud J., Philippon A.-L., Thiebaud P.-C. (2025). Impact of virtual, augmented or mixed reality in basic life support training: a scoping review. Clin Simul Nurs.

[12] Sun R., Wang Y., Wu Q., Wang S., Liu X., Wang P. (2024). Effectiveness of virtual and augmented reality for cardiopulmonary resuscitation training: a systematic review and meta-analysis. BMC Med Educ.

[13] Trevi R., Chiappinotto S., Palese A., Galazzi A. (2024). Virtual reality for cardiopulmonary resuscitation healthcare professionals training: a systematic review. J Med Syst.

[14] Van Merrienboer J.J., Sweller J. (2005). Cognitive load theory and complex learning: recent developments and future directions. Educ Psychol Rev.

[15] Militello L.G., Sushereba C.E., Ramachandran S. (2023).

[16] Hart S.G., Staveland L.E. (1988).

[17] Kirkpatrick D., Kirkpatrick J. (2006).

[18] Kirkpatrick J.D., Kirkpatrick W.K. (2016).

[19] Eldridge S.M., Chan C.L., Campbell M.J., Bond C.M., Hopewell S., Thabane L. (2016). CONSORT 2010 statement: extension to randomised pilot and feasibility trials. BMJ.

[20] Hopewell S., Chan A.-W., Collins G.S., Hróbjartsson A., Moher D., Schulz K.F. (2025). CONSORT 2025 statement: updated guideline for reporting randomised trials. Lancet.

[21] Goldsworthy A., Olsen M., Koh A., Demaneuf T., Singh G., Almheiri R. (2024). Extended reality head-mounted displays are likely to pose a significant risk in medical settings while current classification remains as non-critical. Microorganisms.

[22] Hernández-Padilla J., Suthers F., Fernández-Sola C., Granero-Molina J. (2016). Development and psychometric assessment of the basic resuscitation skills self-efficacy scale. Eur J Cardiovasc Nurs.

[23] Studnicka K., Zarzycka D., Zalewski J. (2023). Student satisfaction and self confidence in learning scale (SSCL)—reliability and validity test of the Polish version. Signa Vitae.

[24] Braun V., Clarke V. (2006). Using thematic analysis in psychology. Qual Res Psychol.

[25] Braun V., Clarke V. (2021). One size fits all? What counts as quality practice in (reflexive) thematic analysis?. Qual Res Psychol.

[26] O’Brien B.C., Harris I.B., Beckman T.J., Reed D.A., Cook D.A. (2014). Standards for reporting qualitative research: a synthesis of recommendations. Acad Med.

[27] Hou L., Dong X., Li K., Yang C., Yu Y., Jin X. (2022). Comparison of augmented reality-assisted and instructor-assisted cardiopulmonary resuscitation: a simulated randomized controlled pilot trial. Clin Simul Nurs.

[28] Hou L., Dong X., Li K., Yang C., Yu Y., Jin X. (2022). Effectiveness of a novel augmented reality cardiopulmonary resuscitation self-training environment for laypeople in China: a randomized controlled trial. Interdiscip Nurs Res.

[29] De La Vega S.B., Guerif-Dubreucq E., Bouaoud J., Awad M., Mathon L., Beauvais A. (2025). Mixed reality versus manikins in basic life support simulation-based training for medical students in France: the mixed reality non-inferiority randomized controlled trial. J Educ Eval Health Profess.

[30] Li X., Yin X., Huang G., Wang X. (2025). Effectiveness of extended reality technologies in cardiopulmonary resuscitation training: a bayesian network meta-analysis. BMC Emerg Med.

[31] Fijačko N., Rios M.P., Semeraro F., Nadkarni V.M., Greif R. (2025). Resuscitation education science meets virtual and augmented reality: evolution from potential concept to recommendations. Resusc Plus.

[32] Donoghue A.J., Auerbach M., Banerjee A., Blewer A.L., Cheng A., Kadlec K.D. (2025). Part 12: Resuscitation education science: 2025 American Heart Association guidelines for cardiopulmonary resuscitation and emergency cardiovascular care. Circulation.

[33] Nabecker S., de Raad T., Abelairas-Gomez C., Breckwoldt J., Chakroun-Walha O., Farquharson B. (2025). European resuscitation council guidelines 2025 education for resuscitation. Resuscitation.

[34] Artero P.M.A., Rios M.P., Greif R., Cervantes A.B.O., Gijón-Nogueron G., Barcala-Furelos R. (2023). Efficiency of virtual reality for cardiopulmonary resuscitation training of adult laypersons: a systematic review. Medicine.

[35] Finseth T., Lubold N., Goel D., Alcañiz M., Lohre R. (2025).

[36] Ingrassia P.L., Mormando G., Giudici E., Strada F., Carfagna F., Lamberti F. (2020). Augmented reality learning environment for basic life support and defibrillation training: usability study. J Med Internet Res.

[37] Issleib M., Kromer A., Pinnschmidt H.O., Süss-Havemann C., Kubitz J.C. (2021). Virtual reality as a teaching method for resuscitation training in undergraduate first year medical students: a randomized controlled trial. Scand J Trauma Resusc Emerg Med.

[38] Sadeghi A.H., Peek J.J., Max S.A., Smit L.L., Martina B.G., Rosalia R.A. (2022). Virtual reality simulation training for cardiopulmonary resuscitation after cardiac surgery: face and content validity study. JMIR Serious Games.

[39] Lavoie P., Deschênes M.-F., Nolin R., Bélisle M., Garneau A.B., Boyer L. (2020). Beyond technology: a scoping review of features that promote fidelity and authenticity in simulation-based health professional education. Clin Simul Nurs.

[40] Cossio S., Chiappinotto S., Dentice S., Moreal C., Magro G., Dussi G. (2025). Cybersickness and discomfort from head-mounted displays delivering fully immersive virtual reality: a systematic review. Nurse Educ Pract.

