# Supplementary material for: Collocated mixed reality for basic life support training in medical students: a randomised pilot feasibility trial
Source: Resusc Plus. 2026 Jun 10;30:101383. doi: 10.1016/j.resplu.2026.101383 (PMC13312584; doi:10.1016/j.resplu.2026.101383)
Supplement: Online Resource 1 — Preliminary usability, acceptability, and implementation themes derived from open-ended feedback in the MR arm, including subthemes, codes, interpretive summaries, representative quotations with anonymised participant identifiers, and the open-ended feedback prompt. [file mmc1.docx]

Online Resource 1. Preliminary themes related to usability, acceptability, and implementation derived from open-ended feedback in the mixed-reality (MR) arm (n = 10; translated from Hebrew)

| Theme | Subtheme | Codes | Interpretive summary | Representative quotation (participant ID) |
| --- | --- | --- | --- | --- |
| Technical and experiential refinement | Stability during compressions | headset slippage; fit during physical activity | Headset movement during chest compressions was identified as a practical ergonomic issue requiring refinement. | “The VR glasses kept slipping off during resuscitation.” (MR-01) |
|  | Visual and contextual realism | visual realism; contextual cues; background sounds | Participants highlighted the importance of visual and contextual realism in strengthening the simulation experience. | “It would be better if the body looked more realistic and if background sounds were added to the simulation, so that the situation would feel more real.” (MR-02) |
| Perceived added value/usefulness | Added value compared with previous CPR training | experiential value; comparison with prior training; additional supervised practice | Some participants perceived the training as more experiential than previous resuscitation instruction and requested additional practice time. | “The activity was amazing, experiential, and very instructive - a level above the resuscitation course I completed in the past. I would allow a little more time for practice with professionals.” (MR-03) |
|  | Potential if more interactive | avatar responsiveness; interactivity; contingent reactions | Participants identified greater interactivity as an important target for future MR development. | “Virtual reality could be amazing if it were interactive.” (MR-04) |
|  | Avatar response and physical alignment | avatar reactions; alignment with manikin; clarity of augmentation | MR-specific feedback indicated that responsiveness and alignment should be refined together. | “The VR could be cool if there were responses from the avatar in the future and if it aligned well with the physical manikin; otherwise, it is a little unclear.” (MR-05) |
|  | Mixed views on the added benefit | limited added value; unnecessary headset; good refresher | Not all participants perceived a clear added benefit from the MR layer. | “I did not feel that the glasses added much. Other than that, it was a good refresher.” (MR-06) |
| Integration into teaching | Experiential learning | experiential simulation; instructional value; lecture pacing | Participants valued experiential practice, while some also identified the need to improve the preceding instruction. | “The lecture felt quite rushed. The simulation was experiential and instructive.” (MR-07) |
|  | Small groups and practice | small-group learning; opportunity to practise; opportunity to ask questions | Small-group practice was viewed as supporting active participation and questions. | “It was good that we were a small group; it helped us practise and ask questions. The physical experience is, of course, very important.” (MR-08) |
|  | Desire for more practice over time | longitudinal practice; maintenance of competence; confidence | Participants expressed interest in opportunities for repeated practice to maintain skills and confidence. | “It was excellent. In my opinion, it would be worthwhile to practise on the manikins in our faculty emergency room periodically, in order to maintain our competence and confidence in providing assistance.” (MR-09) |
| Overall impressions | Overall positive impression | positive overall experience; additional independent practice | Some feedback reflected a positive overall impression while identifying opportunities for additional independent practice. | “It was excellent; more independent practice would be nice.” (MR-10) |

*Note: Quotations were translated from Hebrew by the research team. Minor wording adjustments were made for readability while preserving the original meaning. Participant identifiers are anonymised. Participants occasionally used the terms “VR” and “VR glasses” in their responses; these terms were retained in the translated quotations. The open-ended prompt was administered to participants in both study arms; the table presents responses from participants in the MR arm.*

Open-ended feedback prompt:

Participants in both study arms were invited to respond to the following open-ended prompt immediately after training:

“We would appreciate hearing any comments, insights, or personal feedback you have about the training, including what was particularly helpful, what could be improved, and how you felt during the process.”
