# Supplementary material for: Collocated mixed reality for basic life support training in medical students: a randomised pilot feasibility trial
Source: Resusc Plus. 2026 Jun 10;30:101383. doi: 10.1016/j.resplu.2026.101383 (PMC13312584; doi:10.1016/j.resplu.2026.101383)
Supplement: Online Resource 2 — Situational Motivation in Clinical Training scale (SMCIT). [file mmc2.docx]

# Participant Questionnaire – Training Day (Before Instruction Begins)

**Pre-Training Motivation for CPR Learning (SMCIT–Pre)**

This section asks how motivated you feel to learn and engage in the CPR training.

Please indicate your level of agreement with each statement by choosing a number from the scale below:

| **No.** | **Statement** | **Strongly disagree (1)** | **Disagree (2)** | **Neutral (3)** | **Agree  (4)** | **Strongly agree (5)** |
| --- | --- | --- | --- | --- | --- | --- |
| 1 | I feel motivated to improve my CPR skills. | **1** | **2** | **3** | **4** | **5** |
| 2 | I believe I will enjoy learning CPR using the training method provided. | **1** | **2** | **3** | **4** | **5** |
| 3 | I would prefer to learn other clinical skills using similar methods (e.g., simulation or VR). | **1** | **2** | **3** | **4** | **5** |

**Participant Questionnaire – Post Training**

**Post-Training Motivation for CPR Learning (SMCIT–Post)**

Please indicate your level of agreement with each statement by choosing a number from the scale below:

| **No.** | **Statement** | **Strongly disagree (1)** | **Disagree (2)** | **Neutral (3)** | **Agree  (4)** | **Strongly agree (5)** |
| --- | --- | --- | --- | --- | --- | --- |
| 1 | The training increased my motivation to continue improving my CPR skills. | **1** | **2** | **3** | **4** | **5** |
| 2 | I would prefer to learn additional topics using a similar method (e.g., simulation or VR). | **1** | **2** | **3** | **4** | **5** |
