# Supplementary material for: Collocated mixed reality for basic life support training in medical students: a randomised pilot feasibility trial
Source: Resusc Plus. 2026 Jun 10;30:101383. doi: 10.1016/j.resplu.2026.101383 (PMC13312584; doi:10.1016/j.resplu.2026.101383)
Supplement: Online Resource 3 — Participant flow diagram. [file mmc3.docx]

Online Resource 3. Participant flow diagram.


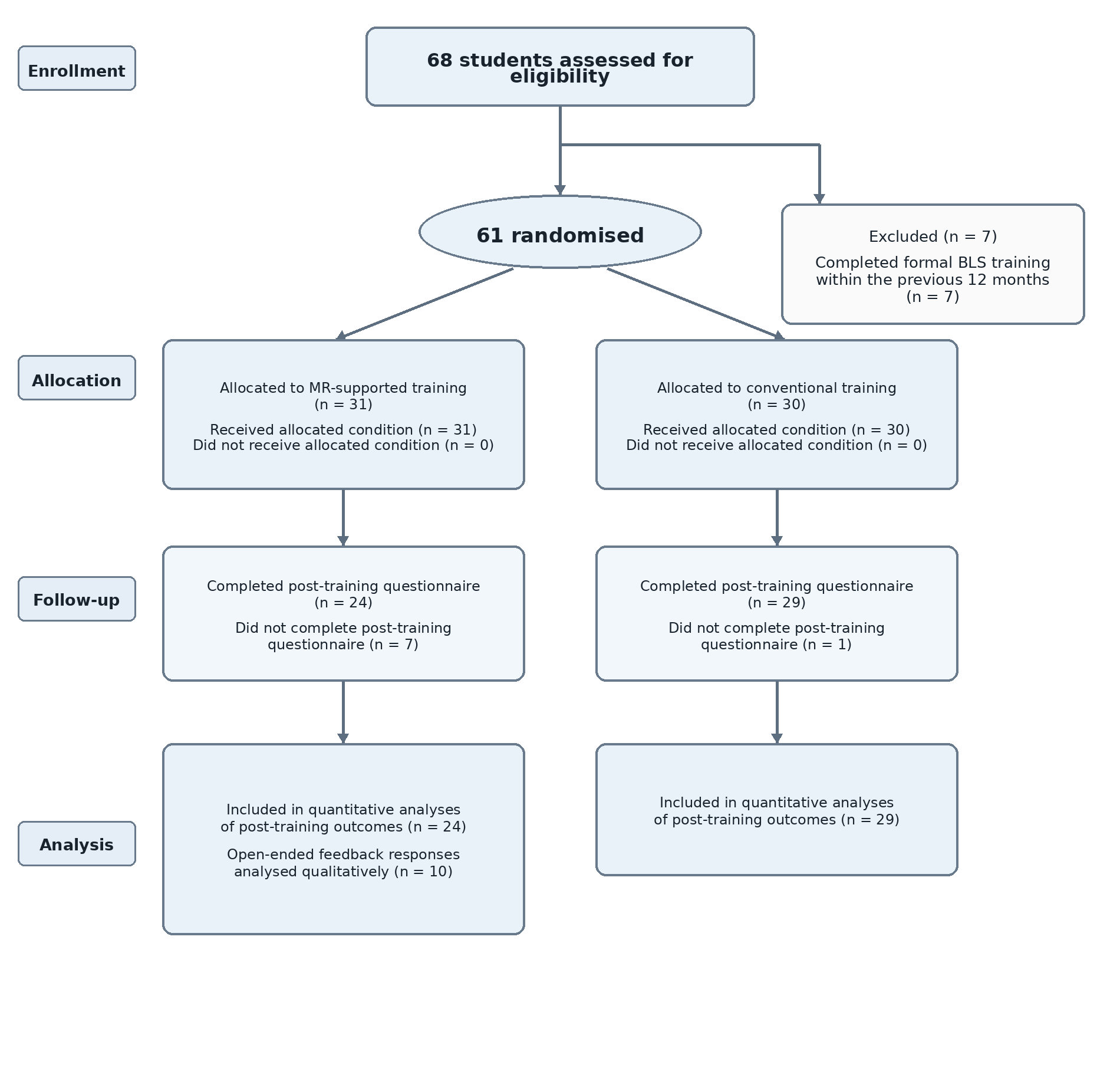


*BLS = basic life support; MR = mixed reality. Of the 68 students assessed for eligibility, 7 were excluded because of formal BLS training within the previous 12 months. The remaining 61 students provided informed consent, were randomised, and received their allocated training condition: 31 were allocated to MR-supported training and 30 to conventional training. Post-training questionnaires were completed by 24 participants in the MR-supported arm and 29 participants in the conventional arm. Ten participants in the MR-supported arm provided open-ended feedback that was included in the exploratory qualitative analysis.*

*.*
